# The central nervous system of sea cucumbers (Echinodermata: Holothuroidea) shows positive immunostaining for a chordate glial secretion

**DOI:** 10.1186/1742-9994-6-11

**Published:** 2009-06-18

**Authors:** Vladimir S Mashanov, Olga R Zueva, Thomas Heinzeller, Beate Aschauer, Wilfried W Naumann, Jesus M Grondona, Manuel Cifuentes, Jose E Garcia-Arraras

**Affiliations:** 1 Department of Biology, University of Puerto Rico, P.O. Box 23360, UPR Station, Rio Piedras, PR 00931-3360, Puerto Rico; 2 Anatomische Anstalt, Ludwig-Maximilians-Universität München, Pettenkoferstrasse 11, D-80336 München, Germany; 3 Institut für Zoologie, Universität Leipzig, Talstrasse 33, D-04103 Leipzig, Germany; 4 Laboratorio de Fisiología Animal. Departamento de Biología Celular, Genética y Fisiología. Facultad de Ciencias. Universidad de Málaga. 29071 Málaga. Spain

## Abstract

**Background:**

Echinoderms and chordates belong to the same monophyletic taxon, the Deuterostomia. In spite of significant differences in body plan organization, the two phyla may share more common traits than was thought previously. Of particular interest are the common features in the organization of the central nervous system. The present study employs two polyclonal antisera raised against bovine Reissner's substance (RS), a secretory product produced by glial cells of the subcomissural organ, to study RS-like immunoreactivity in the central nervous system of sea cucumbers.

**Results:**

In the ectoneural division of the nervous system, both antisera recognize the content of secretory vacuoles in the apical cytoplasm of the radial glia-like cells of the neuroepithelium and in the flattened glial cells of the non-neural epineural roof epithelium. The secreted immunopositive material seems to form a thin layer covering the cell apices. There is no accumulation of the immunoreactive material on the apical surface of the hyponeural neuroepithelium or the hyponeural roof epithelium. Besides labelling the supporting cells and flattened glial cells of the epineural roof epithelium, both anti-RS antisera reveal a previously unknown putative glial cell type within the neural parenchyma of the holothurian nervous system.

**Conclusion:**

Our results show that: a) the glial cells of the holothurian tubular nervous system produce a material similar to Reissner's substance known to be synthesized by secretory glial cells in all chordates studied so far; b) the nervous system of sea cucumbers shows a previously unrealized complexity of glial organization. Our findings also provide significant clues for interpretation of the evolution of the nervous system in the Deuterostomia. It is suggested that echinoderms and chordates might have inherited the RS-producing radial glial cell type from the central nervous system of their common ancestor, i.e., the last common ancestor of all the Deuterostomia.

## Background

According to both traditional and recent molecular phylogenies, the Deuterostomia constitutes a monophyletic supertaxon, which includes three phyla Chordata, Hemichordata and Echinodermata. Echinoderms and hemichordates are grouped together into the clade Ambulacraria, which is treated as a sister group to the chordate lineage [1-4]. Despite the recent progress in molecular phylogenetic analyses, developmental biology, and paleontological discoveries, the mystery of deuterostomian evolution is still far from being fully resolved. In part, this may be due to the need of revisiting macro- and microscopic anatomy of some of the basal groups using standard state-of-the-art morphological techniques. For instance, to interpret the growing body of data on gene expression patterns in echinoderms and hemichordates, the scholars often have to rely upon excellent, but largely outdated, descriptions, which had been published even before electron microscopy came into wide use [5-7]. Since the fierce nineteenth-century debate between Geoffroy Saint-Hilaire and Georges Cuvier, the organization of the nervous system has been one of the foremost criteria in understanding and comparing the body plans of multicellular animals. The potential of the nervous system to provide a wealth of valuable phylogenetic clues has been also emphasized in recent gene expression pattern studies [6-9].

Echinoderms have often been referred to as highly derived and, therefore, considered of limited or no importance for reconstructing the phylogenetic history of the Deuterostomia [8,10]. However, in spite of the set of peculiar features that characterize the phylum (such as pentaradial symmetry in extant forms, mesodermal calcareous endoskeleton, a unique water-vascular system of coelomic nature), recent studies have shown that there may be more common traits between echinoderms and chordates, than was previously thought [11-15]. The phylum Echinodermata is the only non-chordate deuterostomian group that has a centralized nervous system (CNS), represented in these animals by a circumoral nerve ring and (usually five) radial nerve cords. One of the most intriguing common traits shared by the central nervous system of chordates and echinoderms is the presence of a non-neural cell type that is well defined by prominent bundles of intermediate filaments in the cytoplasm and an elongated shape that allows the cells to span the whole thickness of the neural parenchyma. In all vertebrates studied so far, this cell type, termed radial glia, plays a crucial role in histogenesis of the CNS (reviewed by [16]). They serve as intermediate precursors between the so-called neuroepithelial cells that form the wall of the undifferentiated neural tube and the following differentiated progeny. In echinoderms, similar radial glia-like cells appear in the nervous tissue soon after the anlage of the adult nervous system is established in an early juvenile [17] and remain the key non-neural component of the radial nerve cords and the nerve ring in the adult animal [11,14,18]. There is no data on the role of these cells in histogenesis of the nervous tissue in normal development or adult neurogenesis, but it has been recently shown that, at least in sea cucumbers, they do contribute significantly to the post-traumatic regeneration by guiding the migration of neurons and giving rise to new neurons and glial cells in the regenerating nervous system [15].

In chordates, radial glial cells of certain regions of the nervous system are known to produce and secrete the so-called Reissner's substance (RS) [19-21]. The main component of RS is the glycoprotein known as SCO-spondin [22]. The exact function of this protein still remains to be determined, but analysis of its amino acid sequence suggests that it may act as an extracellular modulator of cell adhesion, that may be important for cell migration and axonal guidance [22,23]. This hypothesis is supported by in vitro experiments showing that SCO-spondin can trigger an increased sprouting and neurite outgrowth, as well as neuronal aggregation [24,25].

The main source of RS in vertebrates is the subcomissural organ located in the roof of the third ventricle of the brain, at the entrance to the Sylvian aqueduct. It is composed of secretory radial glial cells that differentiate early in embryogenesis but persist into adulthood and maintain the ability to produce and release their secretion in mature animals [26,27]. The Reissner's substance is also transiently produced and secreted early in embryogenesis by radial glia of the floor plate [28-30].

The RS-producing secretory glial cell type seems to be a taxonomically widespread and phylogenetically conserved trait. Their presence has been confirmed in all major chordate taxa, ranging from human to cephalochordates [19,31-33]. Moreover, supporting radial glia-like cells of the ectoneural radial cords of an echinoderm, the sea star *Asterias rubens*, were shown to be able to produce and secrete RS-like material [11]. The ability of echinoderm glial cells to produce RS is of particular interest not only from the evolutionary point of view, but also because echinoderms can rapidly repair injured nervous tissue and RS is known to be involved in neuronal aggregation and neurite outgrowth. However, since the sea star is the only echinoderm with the documented presence of RS in the nervous system, there can be a problem. The extant echinoderms display two basic designs of their central nervous system. Asteroids and crinoids, are characterized by the superficial localization of the ectoneural radial cords and the nerve ring, which are integrated in the epidermis of the oral body surface [11,34,35]. In the remaining three classes, holothurians, brittle stars, and echinoids, the ectoneural system is internalized to form a subepidermal hollow nerve tube [13,14,18]. It has been convincingly shown that the production and secretion of RS is not unique to the secretory glial cells of the CNS. In planarians, for instance, strongly immunoreactive secretory cells are located in the epidermis. Certain vertebrate epidermal cells are also reported to be positively labeled with anti-RS antibodies [36]. Although the ectoneural cords of the sea star belong to the central nervous system, they are actually still a part of the epidermis. Therefore, it remains unclear whether the RS-like material plays some specific role(s) in the nervous system of the sea star, or it is produced and secreted by the gliocytes just to contribute to the functions performed by the epidermis. A related question is whether the radial glia-like supporting cells of the ectoneural neuroepithelium still retain the ability to produce RS in those echinoderms, whose tubular ectoneural cords have lost their connection with the epidermis and, vice versa, whether the epidermis devoid of the radial nerve cord is still able to produce this material independently of glial cells.

In this study, we describe RS-like immunoreactivity in sea cucumbers, echinoderms with a tubular nervous system, which has been recently emerging as a valuable model system used to resolve some controversial issues in organization, histogenesis, and post-traumatic regeneration of the echinoderm nervous system [14,15,17,37].

## Results

### Nervous system

**Figure 1:**
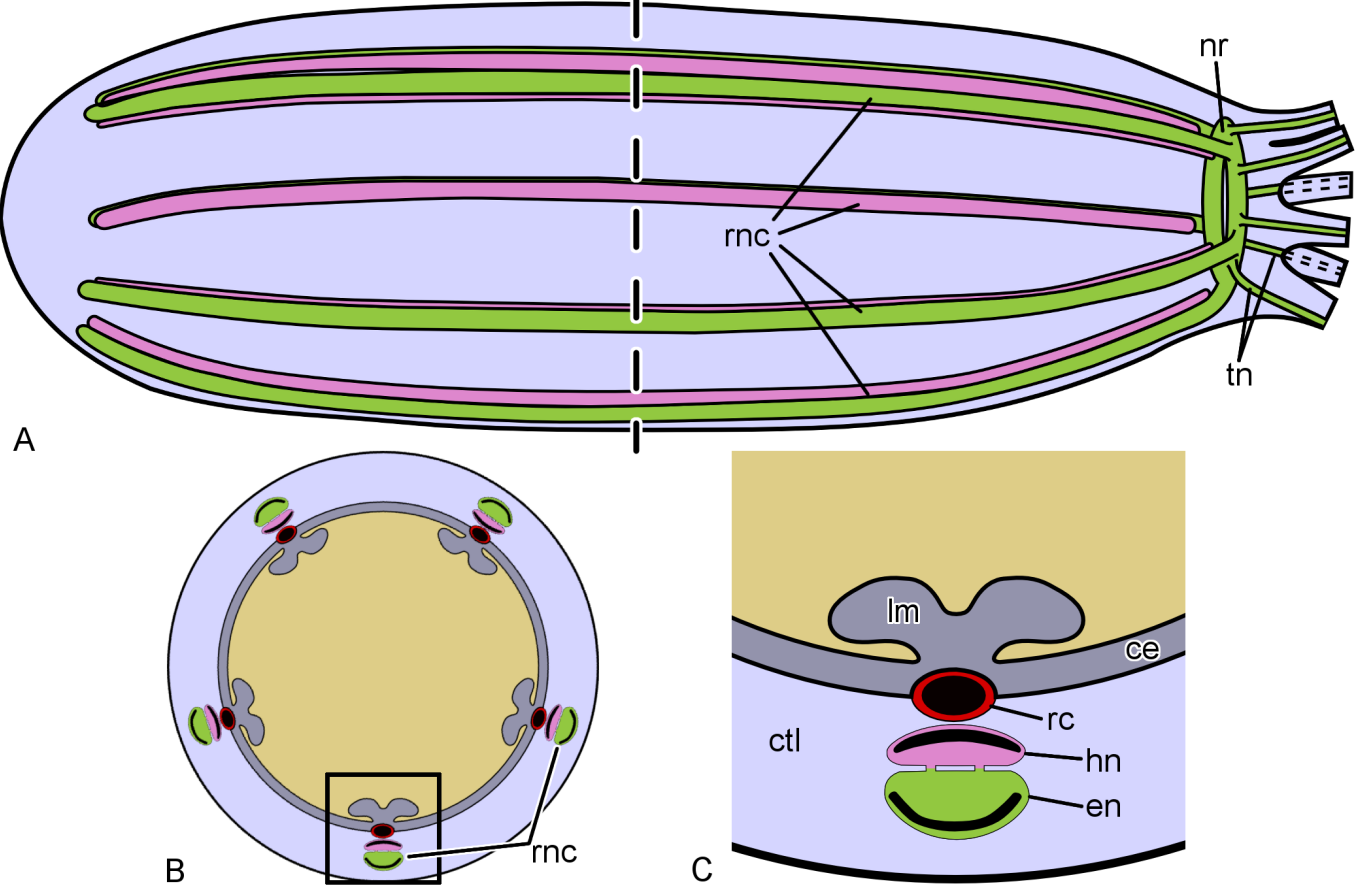
**Simplified diagrams of the anatomical organization of the holothurian nervous system**. **A**. Lateral view of an adult sea cucumber, oral end to the right. The dashed line corresponds to the cross section at the mid-body level shown in **(B)**. **C**. Higher magnification of the boxed area in (**B**) showing the relationship between the radial nerve cords and other radial organs. ce, coelomic epithelial lining of the body cavity; ctl, connective tissue layer of the body wall; en, ectoneural part of the radial nerve cord; hn, hyponeural part of the radial nerve cord; lm, longitudinal muscle band; nr, nerve ring; rc, radial canal of the water-vascular system; rnc, radial nerve cord; tn, tentacular nerve. Not to scale.

**Figure 2:**
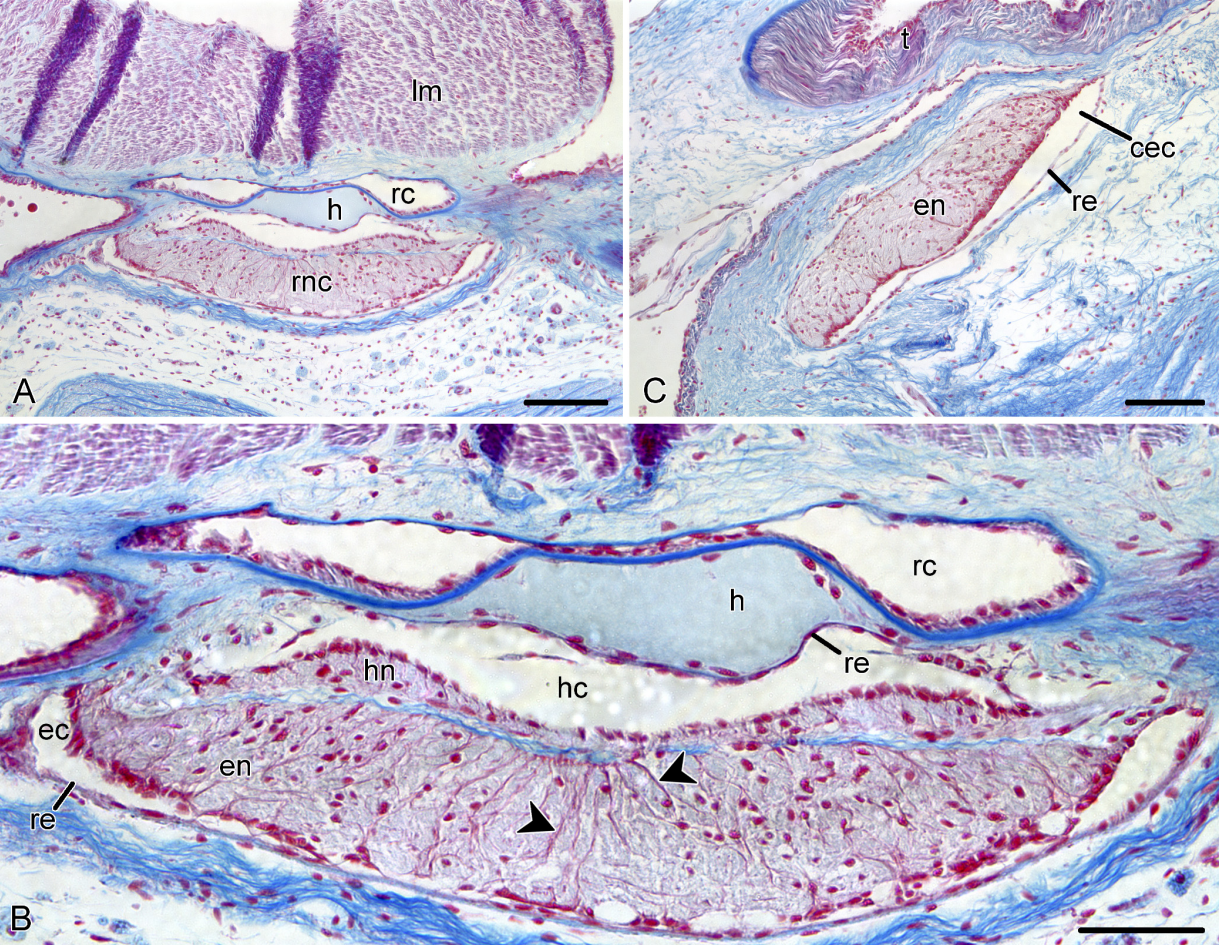
**Overview of the central nervous system (*E. fraudatrix*) on paraffin sections stained with Heindenhein's azan**. **A**. Transverse section showing the radial nerve cord (rnc) and other radial organs, including the longitudinal muscle band (lm), radial water-vascular canal (rc), and the hemal lacuna (h). **B**. Higher magnification of the radial nerve cord shown in **(A)**. Basal processes of glial supporting cells can be clearly seen in the neuroepithelia (arrowheads). **C**. Sagittal section through the pharyngeal bulb, showing the nerve ring. en, ectoneural neuroepithelium. The oral end of the animal is to the right. cec, circular epineural canal of the nerve ring; ec, epineural canal; en, ectoneural neuroepithelium; hc, hyponeural canal; hn, hyponeural neuroepithelium; re, non-neural roof epithelium; t, hydrocoelic lining of the tentacle. Scale bars = 100 μm in (**A**) and (**B**), 50 μm in (**C**).

To provide the reader with some background on the organization of the sea cucumber nervous system, a brief overview follows (see references [14] and [34] for more detail). The major components of the nervous system in adult sea cucumbers are a circumoral nerve ring and five radial nerve cords (Fig. 1A). The nerve cords run from the anterior to the posterior end of the body within the inner connective tissue layer of the body wall. They are accompanied by other radial organs, including (from the outside to the inside) the haemal lacuna, a water-vascular canal, and a longitudinal muscle band (Fig. 1, 2A). Each of the radial nerve cords is composed of two closely apposed strips of nervous tissue, the outer ectoneural cord and the inner hyponeural cord (Fig. 1, 2B). The ectoneural part of the radial nerve cord is considerably thicker than the hyponeural band, and the two are interconnected via numerous short neural bridges along the length of the radial nerve. At the anterior end of the body, the ectoneural bands of adjacent radial cords fuse together to form a circumoral nerve ring that lies perpendicular to the main body axis. The nerve ring of sea cucumbers is devoid of the hyponeural component (Fig. 1A, 2C). The histological organization of the ectoneural ring is similar to the organization of the ectoneural part of the radial nerve cord. Both the ectoneural and hyponeural parts of the nervous system are hollow tubular structures, whose cavities are called the epineural and hyponeural canals, respectively (Fig. 2B, C). The lumen of the former is often quite narrow, slit-like, so that it can be difficult to detect it on paraffin or cryostat sections. The hyponeural canal is usually much wider. The inner wall of the epineural canal and the outer wall of the hyponeural canal are thickened to form a tall neuroepithelium. The opposite side of the canals is overlaid by a thin non-neuronal epithelium, the epineural or hyponeural epithelium, respectively.

A distinct feature of both the ectoneural and hyponeural neuroepithelia is the glial framework composed of glial supporting cells, which bear striking similarities to radial glial cells of vertebrates. The cell bodies of these cells lie in the apical region of the neuroepithelium and make contact with the lumen of the epineural or hyponeural canal. The bases of the somata extend to form long basal processes, which penetrate the whole thickness of the underlying parenchyma and anchor to the basal lamina of the neuroepithelium (Fig. 2B). Another characteristic feature of the supporting glial cells is the long bundles of intermediate filaments, which run from the apical region of the cell to the distal end of the basal processes [14,18]. The glial cells that make up the non-neuronal roof epithelia of the epineural and hyponeural canals, although different in shape, are believed to be a variant of radial glia-like supporting cells and, like the latter, also contain bundles of intermediate filaments in their cytoplasm [14].

**Figure 3:**
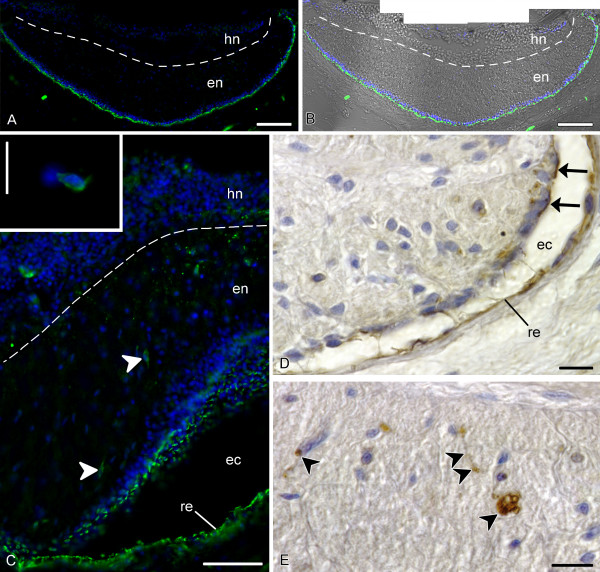
**RS-like immunoreactivity in the ectoneural part of the radial nerve cord, transverse sections**. Dashed line shows the border between the ectoneural (en) and hyponeural (hn) parts of the radial nerve cord. **A, B **Low-magnification view of the radial nerve cord of *H. glaberrima *showing (**A**) Immunofluorescent microscopy with the AFRU antiserum (green) and nuclei stained with Hoechst (blue) and (**B**) the immunofluorescence combined with a Hoffman DIC image. Note the intensely labelled immmunoreactive material overlaying the apical surface of the ectoneural neuroepithelium. **C**. High magnification view of the radial nerve cord of *H. glaberrima *with a locally dilated lumen of the epineural canal (ec). AFRU-positive labelling (green) is clearly associated with the apical surface of the ectoneural neuroepithelim (en) and with the non-neural epineural roof epithelium (re). Note also immunoreactive cell bodies (arrowheads) and dotted labelling in the neural parenchyma. The ***inset ***shows a high magnification view of an immunoreactive cell body within the ectoneural parenchyma. **D, E**. Peroxidase-antiperoxidase immunohistochemistry with the RS-K10 antiserum on paraffin sections of the radial nerve cord of *E. fraudatrix*. **D**. Labelling associated with the apical region of the ectoneural neuroepithleium and with epineural roof epithelium (re). Note the deposition of the immunopositive material on the apical surface of the neuroepithelium (arrows). **E**. Labelled cell bodies (arrowheads) and processes (double arrowheads) within the ectoneural parenchyma. Scale bars = 100 μm in (**A**) and (**B**), 50 μm in (**C**), 10 μm in (**C inset**), (**D**), and (**E**).

In the radial nerve cord (RNC), immunostaining with both anti-RF antibodies produced very strong intracellular and extracellular labelling associated with the apical surface of the ectoneural neuroepithelium and the squamous epineural roof epithelium (Fig. 3A–D). The extracellular immunoreactive material seems to be deposited as a cap-like covering on the apices of the cells (Fig. 3C, D). These individual patches are often seen to fuse together to form a continuous layer on the apical surface of the epithelium. However, the labelled material never aggregates in the form of a morphologically identifiable compact structure within the lumen of the epineural canal. Both antisera also reveal immunoreactive cell bodies with fine cytoplasmic staining, which are scattered singly within the parenchyma of the ectoneural neuroepithelium (Fig. 3C, E). The immunopositive cell bodies vary greatly in shape, from rounded to fusiform or irregular, and often seem to give off one or more processes. There is also scattered dotted labelling in the ectoneural parenchyma (Fig. 3C), the dots being most probably the cross-sectioned cellular processes. Some of the labelled processes lay in the plane of sectioning (Fig. 3E).

**Figure 4:**
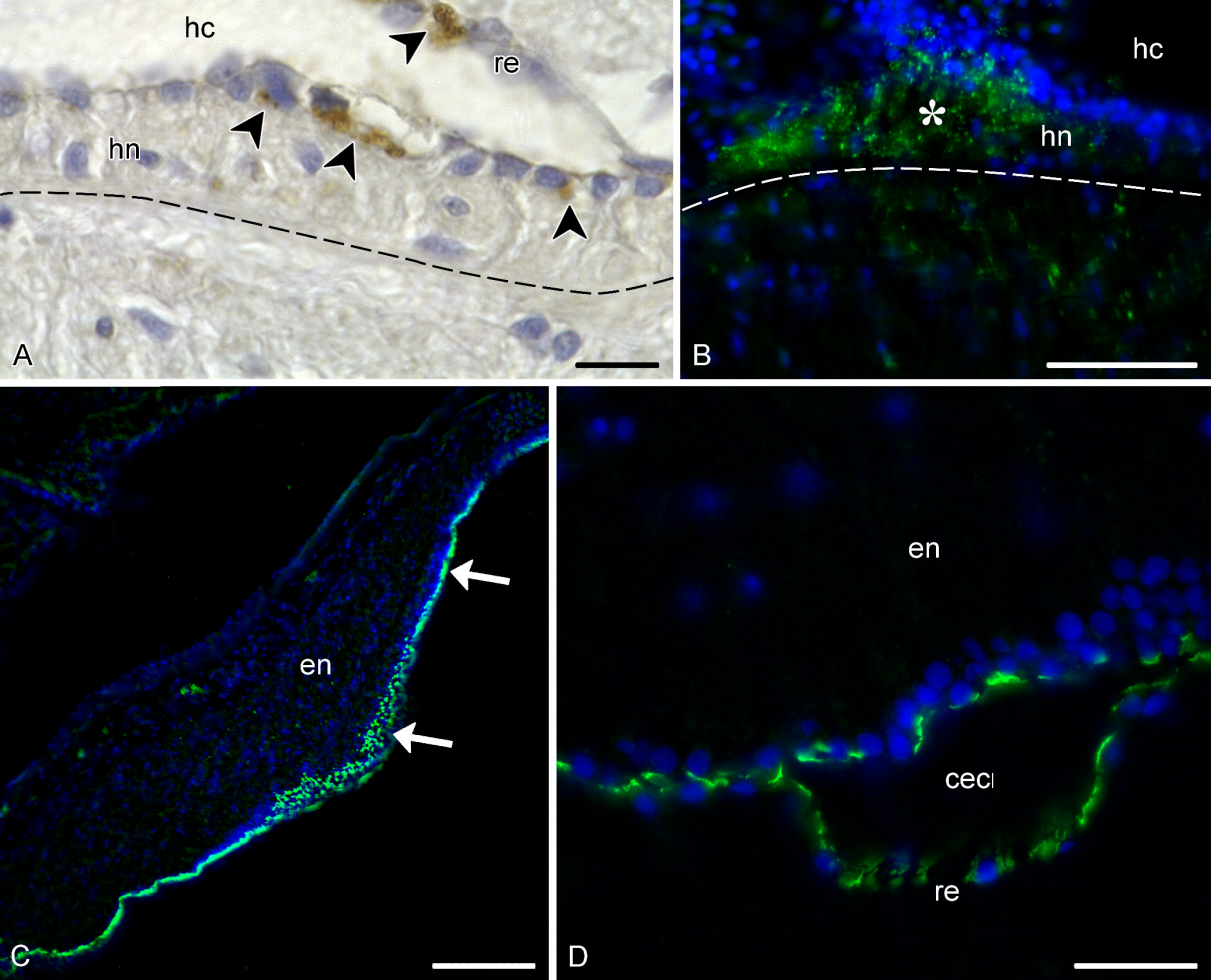
**RS-like immunoreactivity in the hyponeural part of the radial nerve and in the nerve ring**. Dashed line shows the basal border of the hyponeural (hn) part of the radial nerve cord. **A**. Peroxidase-antiperoxidase immunohistochemistry with the RS-K10 antiserum on a transverse section of the radial nerve cord of *E. fraudatrix *showing immunopositive cells (arrowheads) in the hyponeural neuroepithelium (hn) and in the non-neural roof epithelium (re). Note that there is no immunoreactive material neither on the apical surfaces of the epithelia, nor in the lumen of the hyponeural canal (hc). **B**. Dotted labelling (green) in the lateral region of the hyponeural neuroepithelium (asterisk) of *H. glaberrima *visualized by immunostaning with the AFRU antiserum. Nuclei are stained with Hoechst (blue). **C**. Sagittal section through the pharyngeal bulb (the oral end of the animal is to the right) of *H. glaberrima *showing the intense AFRU-positive immunoreactivity (arrows) associated with the apical surface of the ectoneural neuroepithelium (en) and with the epineural roof epithelium. **D**. High magnification view of a region of the nerve ring with a locally dilated lumen of the circular epineural canal (cec) showing the AFRU-positive labelling (green) in the apical region of the ectoneural neuroepithelium and in the epineural roof epithleium (re). Nuclei are stained with Hoechst (blue). Scale bars = 10 μm in (**A**), 50 μm in (**B**), 15 μm in (**C**), 20 μm in (**D**).

The most significant difference in the pattern of RS-like immunoreactivity between the ectoneural and the hyponeural parts of the radial nerve cord is that there is no accumulation of the immunoreactive material on the apical surface of the hyponeural neuroepithelium or the hyponeural roof epithelium (Fig. 4A, B). Although both antisera do label cells in the the hyponeural part of the RNC, the immunopositive cells are less abundant than in the ectoneural part. The two antisera used in the present study show a difference in immunostaining pattern in the hyponeural parenchyma. The RS-K10 antiserum labels singly scattered cell body and processes, as it does in the ectoneural parenchyma (not shown). The AFRU antiserum produces a dotted immunostaining which is much denser than in the ectoneural neuroepithelium. This is particularly evident in the lateral regions of the hyponeural neuroepithelium (Fig. 4B).

The pattern of RS-like immunolabelling in the nerve ring was identical to that in the ectoneural part of the RNC (Fig. 4C, D), i.e. the strongest immunoreactivity is associated with the apical region of the ectoneural epithelium and with the epineural epithelium, although there are also some more moderately labelled cell bodies and processes in the neural parenchyma.

It is interesting to note that, contrary to the data on the basal route of secretion of the RS in chordates [38], neither the ectoneural, nor the hyponeural neuroepithelia of the two sea cucumber species studied showed any significant RS-like immunostaining associated with the basal region.

**Figure 5:**
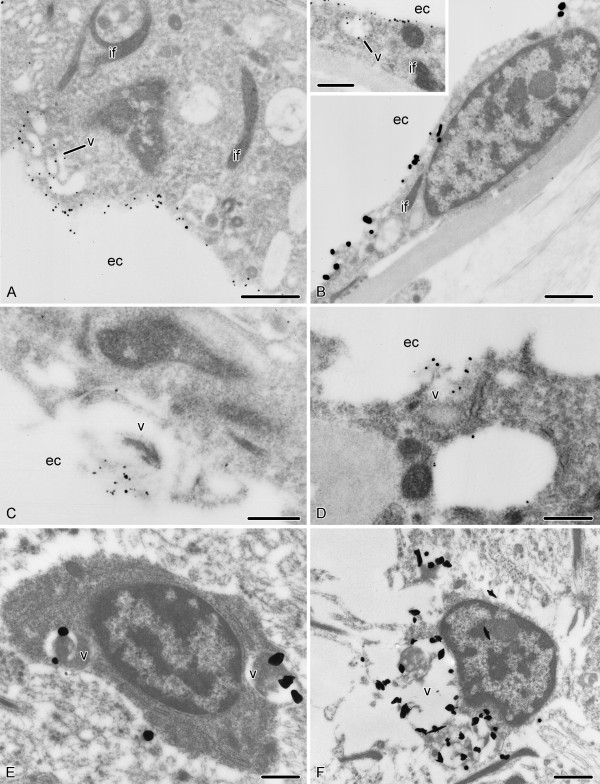
**Ultrastructural immunolocalization of the RS-like material in the holothurian nervous system**. For an explanation of the difference in grain size (smaller grains in **A**, **B **inset, **C **and **D **and larger grains in **B**, **E**, and **F**), refer to the *Methods *section. **A**. Apical surface of a supporting glial cell of the ectoneural neuroepithelium covered with the RS-K10-positive secretion. Note also labelling of the electron-translucent vesicles in the apical cytoplasm of the cell. **B**. RS-K10-positive labelling of the apical surface of the epineural roof epithelium. The ***inset ***shows a labelled vacuole in the cytoplasm of a flattened glial cell. **C, D**. Apparent release of the immunoreactive material from secretory vacuoles into the lumen of the epineural canal by supporting glial cells (**C**) and cells of the roof epithelium (**D**), as revealed by immunogold reactions with the RS-K10 (**C**) and AFRU (**D**) antisera. **E, F**. Immunoreactive cell bodies (RS-K10-positive) within the ectoneural parenchyma. ec, epineural canal; if, bundle of intermediate filaments; v, vacuole. Scale bars = 1 μm in (**A**), (**B**), (**F**), 0.5 μm in (**B inset**), (**C**), (**D**), and (**E**).

To define precisely the localization of the RS-like material to particular cell types and also at the subcellular level, we have implemented immunoelectron microscopy. This approach shows that the intense labelling revealed by the immunoperoxidase and immunofluorescent techniques in the tissues bordering the epineural canal is confined to the supporting glial cells of the ectoneural neuroepithelium and the flattened glial cells of the epineural non-neural roof epithelium (Fig. 5A–D), which can be clearly distinguished from other cell types in the nervous tissue by the presence of prominent bundles of intermediate filaments in their cytoplasm [14]. Within these glial cells, the anti-RS antibodies recognize the content of electron-translucent vacuoles in the apical cytoplasm (Fig. 5A–D). They measure about 0.5 μm, vary in shape from spherical to irregular, and sometimes contain a fine fibrillar or flocculent material, but the labelling is not necessarily associated with it. The vacuoles are occasionally seen to fuse with the apical plasma membrane, and the immunoreactive material is released into the lumen of the epineural canal (Fig. 5C, D). The secreted immunopositive substances seem to remain attached to the cells that produced them forming a thin layer covering the cell apices (Figs. 5A, B). This extracellular labelling on the cell surface is often very strong and is usually more intense than the labelling of the vacuolar content within the cells. However, some glial cells show weak immunostaining on their apical surface.

In agreement with our data obtained with immunofluorescence and immunoperoxidase techniques, the immunogold approach with anti-RF antisera also reveals immunoreactive cells within the neural parenchyma (Fig. 5E, F). These subspherical to irregularly shaped cells are interspersed among the processes of neuronal and glial cells. The elliptical nucleus contains large patches of heterochromatin and prominent nucleolus. The cytoplasm contains mitochondria, cisternae of the rough endoplasmic reticulum, and, most notably, vacuoles with heterogeneous immunoreactive content. These vacuoles seem to vary greatly in size and abundance. When large and numerous, they occupy most of the perinuclear cytoplasm and displace the nucleus to the periphery (Fig. 5F). Occasionally, the vacuoles lie in a very close proximity to the plasma membrane. However, we have not seen convincing signs of the release of the immunoreactive material into the extracellular space. It is important to note that this cell type exhibits no morphological features of supporting glial cells (bundles of intermediate filaments) or neurons (characteristic dense and/or light membrane-bound vesicles).

### Epidermis

**Figure 6:**
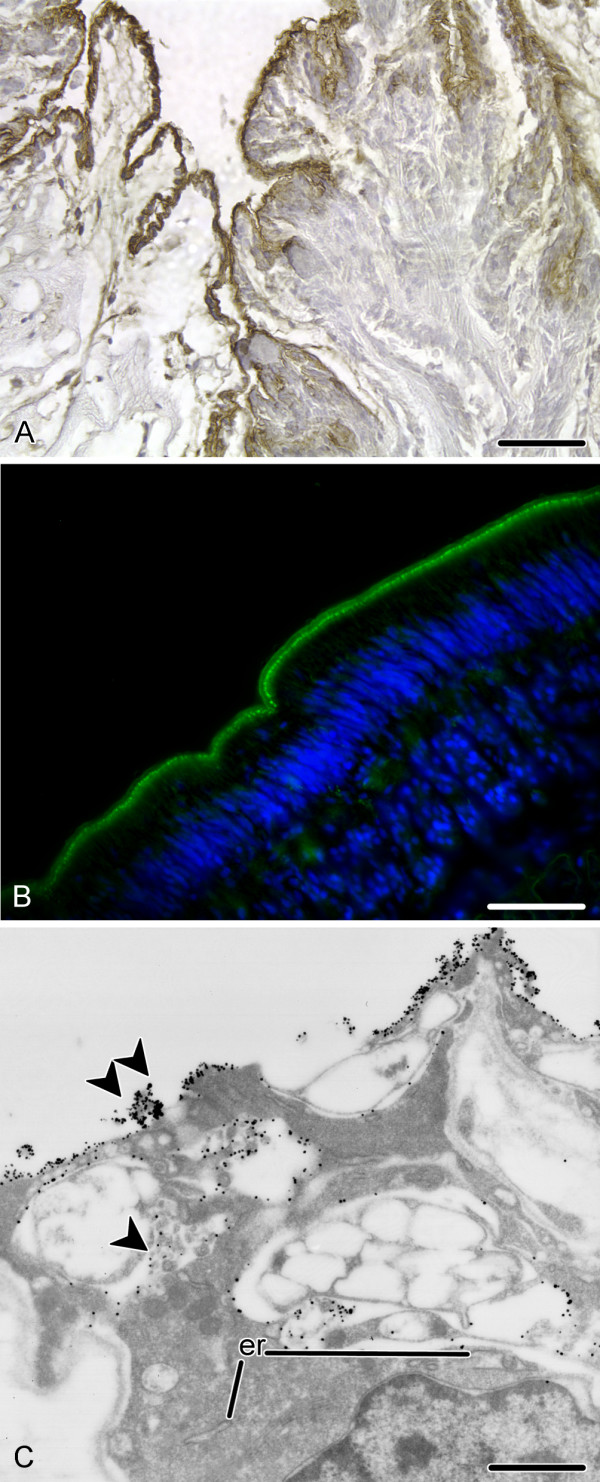
**RS-like immunoreactivity in the epidermis of sea cucumbers**. **A**. Peroxidase-antiperoxidase immunohistochemistry with the RS-K10 antiserum showing strong immunolabelling of the apical region of tentacle epidermis in *E. fraudatrix*. **B**. Immunofluorescent microscopy with the AFRU antiserum reveals intense immunolabelling (green) of the apical surface of the podial epidermis in *H. glaberrima*. Nuclei are stained with Hoechst (blue). **C**. Immunoelectron (RS-K10 antiserum) micrograph of an epidermial cell of *E. fraudatrix *showing strong labelling of the apical surface (double arrowhead) and less prominent labelling of the lateral surfaces (arrowhead) of the cell. er, cisternae of the endoplasmic reticulum. Scale bars = 50 μm in (**A**) and (**B**), 1 μm in (**C**).

Besides specifically recognizing structures within the nervous system, both polyclonal antisera also strongly stain the epidermis (Fig. 6). Immunoreactivity is very intense on the apical surface of the cells, where a thin continuous layer of the immunoreactive material separates the epidermis from the surrounding environment. The lateral and basal surfaces of the cells are also occasionally labelled, but much weaker than the apices (Fig. 6C). Consistent with the immunolabelling of mostly the extracellularly located material, the epithelial cells of the epidermis displayed ultrastructural signs of moderate secretory activity including the presence of cisternae of the endoplasmic reticulum, Golgi apparatus and secretory vacuoles of varying sizes and morphologies.

## Discussion

### Specificity of the immunostaining

The important issue in any immunocytochemical study is the specificity of recognition of the epitope by the primary antibody. The two polyclonal rabbit antisera used in the present study were raised against Reissner's fiber isolated from the bovine spinal cord and a wealth of subsequent studies has convincingly demonstrated that both antisera specifically recognized Reissner's substance (RS) in a wide range of animal species from mammals to planarians [11,19,20,31,36,39,40]. Besides some minor differences in immunolabelling in the hyponeural part of the radial nerve cord, both antibodies used in the present study show similar staining patterns with different visualization techniques employed (peroxidase-anti-peroxidase approach, immunofluorescence, immunoelectron microscopy). The positive immunostaining of holothurian tissues is in agreement with a previous study showing the anti-RS immunoreaction in the ectoneural cord of the sea star *Asterias rubens *[11] and a recent identification of the gene coding for SCO-spondin, the major component of RS, in the genome of the sea urchin *Strongylocentrotus nudus *[41]. The predicted sequence of the protein shows a high degree of conservation of its multidomain structure when compared to the sequences from the Vertebrata.

### Unexpected complexity of glial organization

One of the earliest detailed morphological descriptions of glial supporting cells (Stützzellen) in the nervous system of echinoderms belongs to Bargmann et al., [42]. However, the very relevance of the term glia to the echinoderm nervous system was heavily disputed afterwards [43,44]. This controversy could not be completely resolved upon the basis of merely microscopic observations. Therefore, a need was realized for testing the available reliable markers of glial cells on the echinoderm nervous tissue. In their pioneering work, Viehweg et al. [11] showed that a radial glia-like non-neuronal cell type in the ectoneural system of the sea star *Asterias rubens *is specifically labelled by antibodies raised against bovine Reissner's substance (RS), a phylogenetically conservative secretion produced by glial cells of chordates. In the present study, we demonstrate that two different polyclonal anti-RS antisera reveal three of non-neuronal cell types in the nervous system of sea cucumbers: tall supporting cells in the neuroepithelia, cells of the non-neural roof epithelia, and relatively rare parenchymal cells.

The supporting cells are a very characteristic feature of echinoderm neuroepithelia. They are morphologically similar to radial glial cells of chordates and are readily distinguishable from other cell types due to their elongated shape, perpendicular orientation to the surfaces of the neuroepithelium, and conspicuous bundles of neurofilaments in the cytoplasm [11,14,18,42]. Compared to the starfish, holothurians, and possibly other echinoderms with closed ambulacra, have acquired an additional source of the RS-like substance, the non-neural epineural epithelium that roofs over the ectoneural hyponeural neuroepithelium to seal the epineural canals. The cells of this epithelium are often flattened in shape and, under normal conditions, are never associated with neurons, but are otherwise very similar to the supporting cells of the neuroepithelia and also contain prominent bundles of intermediate filaments in their cytoplasm [14]. The two cell types also display similar patterns of RS-like immunoreactivity. The positively labelled material is seen in the vacuoles, which eventually seem to fuse with the apical plasma membrane and release their content which remains attached to the apical surfaces of the cells. Therefore, our data confirm the observations by Viehweg et al. [11], who showed that the supporting cells of the starfish ectoneural neuroepithelium produce and secrete RS-like material. However, there can be also some differences in the way the RS-like substance is secreted in asteroids and holothuroids. Although the secretory glial cells of the starfish are reported to release the immunopositive both apically and basally, we have observed only apical secretion into the lumen of the epineural canal in both sea cucumber species studied.

Besides the two aforementioned cell types, which have been already known as glial cells in echinoderms [11,14,42], the immunostaining with anti-RS antibodies reveals a previously undescribed cell type within the neural parenchyma of the holothurian CNS. These cells are morphologically distinct from both neurons and supporting cells. They possess neither bundles of intermediate filaments, which are the hallmark of the supporting cells, nor the membrane-bound vesicles that are always seen in neurons. The presence of positively labelled vacuoles in the cytoplasm of these cells, suggest that they are able to produce the RS-like substance. It remains unclear, however, whether they release this material. Although, these parenchymal putative gliocytes account for only about 1% of the total cell population of the radial nerve cord (Mashanov et al., unpublished observation), they are consistently found in different regions of the holothurian CNS. The function of this cell type remains to be determined.

In summary, immunostaining with antibodies raised against Reissner's substance, a phylogenetically conservative secretory product of glial cells in chordates, suggests that (a) the non-neuronal cell types of the echinoderm nervous tissue can be regarded as true glial cells and that (b) the CNS of these non-chordate Deuterostomia comprises several types of glial cells indicating a much higher complexity of neurohistological and functional organization than was thought previously.

### Possible function(s) of the RS-like material in the echinoderm nervous system

Our data suggest that the RS-like material can possibly carry out a variety of functions within a single organism, since it is present in such different tissues as the epidermis and the nervous tissue. These findings coincide with other reports demonstrating the presence of RS and its major component, SCO-spondin, both in the nervous system (e.g., [11,19]) and in non-neural tissues [36,45]. Sequence analysis suggests that SCO-spondin is a multidomain protein with a very complex structure [23,41], which can be involved in a wide range of processes mediated via protein/protein interactions.

The only study of RS-secreting glia in echinoderms published so far [11] deals with the ectoneural system of a sea star, which is integrated in the epidermis of the body wall. The authors demonstrate that the apically secreted immunopositive substance contributes to the formation of the so-called hyalin layer, a film of extracellular material, which separates the epidermis from the environment. Unfortunately, they do not provide any data on RS-like immunoreactivity in other areas of the epidermis lying outside the radial nerve cord. Therefore, it remained unclear whether this positively labelled secretion played some specific role in the nervous system itself or it was produced just as a component of the hyalin layer, as in other regions of the epidermis. In the present study, we demonstrate that holothurian epidermal cells show clear RS-like immunoreactivity. However, strong immunopositive staining is also observed in the submerged ectoneural tube, which has no connection with the epidermis in holothurians. This means that the ectoneural system of echinoderms with closed ambulacra did not lose the ability to produce RS-like material as it detached from the epidermis to give rise to the subcutaneous ectoneural tube in the course of echinoderm evolution. These findings, in turn, imply that the RS-like material does play some important role(s) both in the echinoderm nervous systems and the epidermis. The function of RS in the epidermis is completely unknown [36]. As to the nervous system, RS has been shown to be involved in axonal guidance, as well as in promoting neuronal survival, differentiation, and aggregation in vertebrates [24,30,41]. This neurogenetic role could be of particular interest with respect to known abilities of echinoderms to quickly restore their nervous system after various types of injury [15,34,37,46,47].

RS is also known to be involved in regulation of cerebrospinal fluid homeostasis in vertebrates presumably through regulation of the rate of the cerebrospinal fluid production and/or by preventing the central canal of the tubular CNS from stenosis (reviewed in [48] and [49]). We currently know nothing about the function of the epineural canal in echinoderms. In sea cucumbers, it is usually much narrower than the hyponeural canal, but it is possible that its slit-like lumen should nevertheless remain open for some reason. Therefore, the RS-like material, which forms a nearly continuous film of immunoreactive labelling on the apical surface of the ectoneural neuroepithelium and the roof epithelium, may be involved in preventing the epineural canal from closing.

## Conclusion

A full understanding of the fundamental principles of organization and function of our own nervous system can hardly be achieved without the knowledge of how the human CNS might have evolved. In spite of recent advances in molecular systematics and gene expression pattern studies, there is still no common agreement about the evolution of the deuterostomian body plans in general and of the nervous system in particular. We believe that studying the phylum Echinodermata can be a suitable way to resolve many of the issues in the evolutionary biology of the Deuterostomia. Our results showing that glial cells of the holothurian tubular nervous system produce Reissner's substance, a material released by secretory glial cells in all chordates studied so far [19,39,50], add to the growing body of evidence that there are more parallels between the nervous systems of echinoderms and chordates than was previously thought [11-15]. Possible homology between the ectoneural plate (in crinoids and asteroids) or tube (in echinoids, ophiuroids, and holothurians) of echinoderms and the neural plate/tube of chordates is suggested by a number of morphological and gene expression parallelisms (see [12] and [13] for a review). First, both the ectoneural cord of echinoderms and the neural tube of chordates develop by proliferation of the mid-line subpopulation of ectodermal cells, which in all chordates and cryptosyringid echinoderms (echinoids, ophiuroids, and holothurians) eventually get submerged below the epidermis. The process of internalization of the central nervous system may have evolved independently in the two phyla. Second, development of both the echinoderm ectoneural cords and the chordate neural plate is induced by signal from mesodermal structures (the hydrocoel and notochord, respectively). Third, some of the regulatory genes that organize the chordate neural plate are also expressed in the echinoderm ectoneural cord. One of the most intriguing features shared by both phyla is the presence of a particular non-neuronal cell type in the central nervous system, which is known as radial glia in chordates and supporting glia in echinoderms. In both cases, the cells (a) are very tall and are oriented with their main axis perpendicular to the plane of the neuroepithelium; (b) span the whole thickness of the latter from the apical to basal surface; (c) contain prominent bundles of intermediate filaments in their cytoplasm; (d) play a key role in embryonic and/or post-traumatic neurogenesis; and (e) produce and release a glycoprotein material known as Reissner's substance. Since both echinoderms and chordates possess a centralized nervous system with a distinct RS-producing radial glial cell type, it would be most parsimonious to believe that they both might have inherited those traits from their common ancestor. This implies that the last common ancestor of all the Deuterostomia could have possess a central nervous system (obviously, located in the epidermis, like the ectoneural bands in sea stars and sea lilies) with RS-secreting radial glia-like cells.

## Methods

### Animal sampling and tissue fixation

Adult individuals of *Eupentacta fraudatrix *Djakonov et Baranova, 1958 (Holothuroidea, Dendrochirota) were collected from Vostok Bay, Sea of Japan, in October 2006. Mature individuals of *Holothuria glaberrima *Selenka, 1867 (Holothuroidea, Aspidochirota) in the intertidal zone of the rocky northeast coast of Puerto Rico in October 2006 and December 2008. Pharyngeal bulbs and small pieces of the body wall containing the complex of radial organs were immersion fixed overnight at 4°C (see below). After fixation, the samples were either processed further or stored in the respective buffer until needed (up to 1 month).

### Polyclonal antisera

The rabbit antiserum AFRU was raised against bovine Reissner's fiber extracted in the medium containing urea (0.1 M Tris-HCl pH8.6, 1 mM EDTA, 0.01 M DTT, 8 M urea) [19]. RS-K10 rabbit polyclonal antiserum was obtained using bovine Reissner's substance as the immunogen, but the extraction was performed in a different medium: 0,05 M PBS containing 0,14 M NaCl [26]. Both antisera have been used extensively previously, and have been shown to specifically recognize Reissner's substance in a wide range of diverse species (see Discussion).

### Immunoperoxidase histochemistry

For peroxidase-antiperoxidase (PAP) immunohistochemistry, the tissue samples were fixed in (i) 4% paraformaldehyde, 0.1% glutaraldehyde, 0.15% picric acid in 0.1 M cacodylate buffer (pH 7.6) adjusted to 1090 mOsm and in (ii) 4% paraformaldehyde and 0.1% glutaraldehyde in 0.05 M PBS containing 0.14 M NaCl. The tissues were then decalcified in 25% EDTA (pH 7.4) [51] and embedded in paraffin. Sections (7 μm) were cut with a sliding microtome (Reichert), mounted on slides, re-hydrated and subjected to quenching of endogenous peroxidase activity with 3% H_2_O_2 _for 10 min. The immunostaining was performed using Histostain Plus Kit (Zymed/Invitrogen) following manufacturer's protocol. The first antibodies, AFRU and RS-K10, were applied at a dilution of 1:2000 for 24 to 48 h at 4°C. The immunostaining was visualized using diaminobenzidine (Sigma) as a chromogen. The nuclei were slightly counterstained with Meyer's hematoxylin. The sections were dehydrated and mounted in DPX (Fluka).

For general morphology studies, some of the paraffin sections were stained with Heidenhain's azan (azocarmine, aniline blue, and orange G) [51].

### Transmission immunoelectron microscopy

For immunoelectron microscopy, the fixation was the same as for PAP immunohistochemistry. Pre-embedding labelling technique was used to localize the antigen at the ultrastructural level. After washes in the respective buffer and cryoprotection in 10%, 20%, and 30% buffered sucrose, the samples were frozen in the cryoembedding OCT medium (Sakura) and stored at -25°C until cryostat sectioning (10 μm). Sections were applied onto gelatin-coated slides, dried overnight at 45°C and then stored at -25°C. When needed, the slides were equilibrated at room temperature and washed in PBS. In order to minimize nonspecific antibody binding, the sections were preincubated in 0.05 M buffered glycine followed by 0.1% BSA-c™ (Aurion). The primary antibodies were applied at dilutions ranging from 1:1000 to 1:2000 for 24–48 h at 4°C. After thorough washing, the sections were incubated in a secondary gold-conjugated goat-anti-rabbit antibody (the average gold cluster diameter below 0.8 nm) (Aurion) and briefly post-fixed in 2% glutaraldehyde and 1% osmium tetroxide. To facilitate observation of ultra-small gold clusters, silver enhancement reagents R-Gent SE-EM and R-Gent SE-LM (Aurion), which produce smaller and larger grains, respectively, were used following the protocol, provided by the manufacturer. The sections were then dehydrated in ethanol and embedded in Araldite (Serva). Ultrathin sections (50–70 nm) were cut with glass knives using an Ultracut E (Reichert) ultramicrotome, collected on coated slot grids, and slightly counterstained with a saturated uranyl acetate solution in methanol, and examined and photographed in a Zeiss EM 10 transmission electron microscope.

### Immunofluorescent histochemistry

For immunofluorescent microscopy, the tissues were fixed with 4% paraformaldehyde in 0.01 M PBS (pH 7.4, 1030 mOsm). Frozen sections were obtained as described above. Slides were incubated in PBS containing 0.5% Triton X-100. Autofluorescence was reduced by treating the sections with 0.1 M glycine in PBS. The specimens were then incubated for 1 hour in PBS containing 2% of normal goat serum. The primary anti-RS antisera were applied overnight (4°C) at a dilution of 1:2000 or 1:4000. After rinsing, the slides were incubated in FITC-conjugated goat-anti-rabbit antibodies (1:100) (Biosource) for 1 h at room temperature. Nuclei were visualized with Hoechst 33342. The sections were mounted in buffered glycerol (pH 8.5), viewed and photographed with a Nikon Eclipse 600 microscope equipped with a Spot RT3 digital camera (Diagnostic Instruments, Inc.). Post-acquisition image processing including composite image generation, brightness/contrast adjustments, multi-panel figure assembling, and lettering was performed with public domain programs ImageJ Fiji (available at ) and GIMP 2.6.1 (available at ).

**Figure 7:**
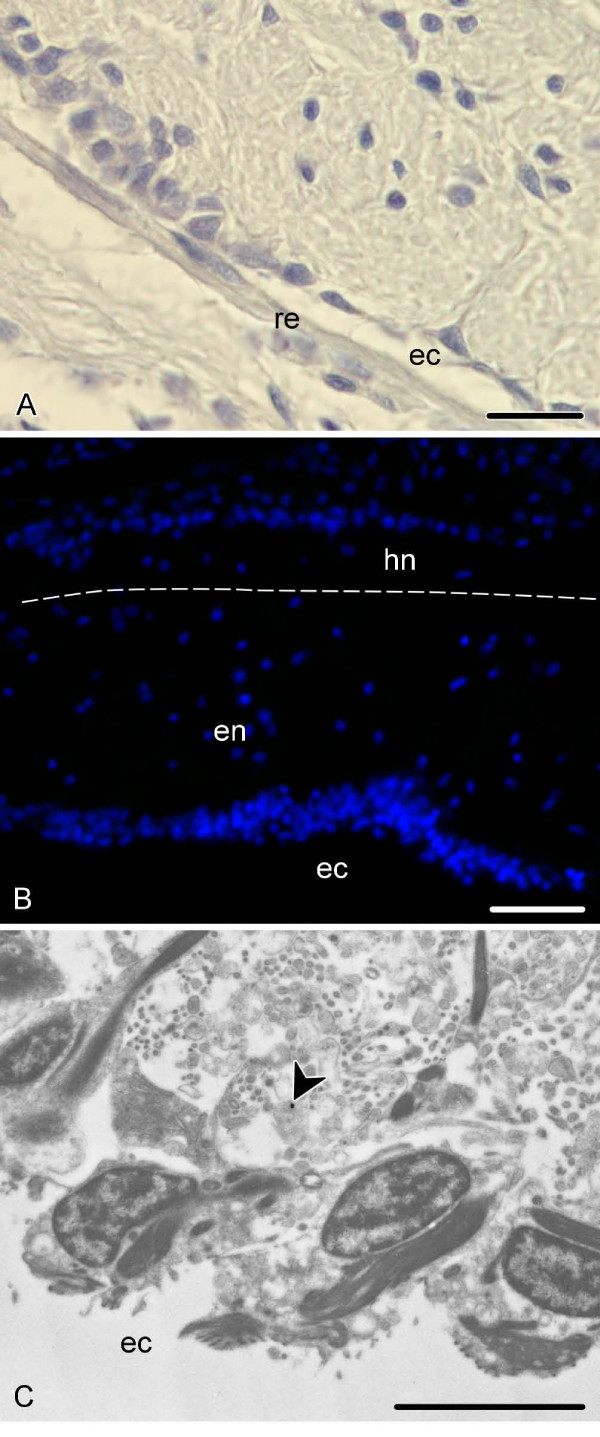
**Negative control sections (see *Methods*)**. **A**. Peroxidase-antiperoxidase immunocytochemistry, transverse paraffin section of the radial nerve cord of *E. fraudatrix*. **B**. Immunofluorescent microscopy; transverse cryosection of the radial nerve cord of *H. glaberrima*. Nuclei are stained with Hoechst (blue). Dashed line shows the border between the ectoneural (en) and hyponeural (hn) parts of the radial nerve cord. **C**. Immunoelectron microscopy; the apical region of the ectoneural neuroepithelium of the radial nerve cord (*E. fraudatrix*). Note a single grain representing unspecific staining (arrowhead). ec, epinueral canal; re, non-neuronal roof epithelium. Scale bars = 20 μm in (**A**), 50 μm in (**B**), 5 μm in (**C**).

Controls were performed by substituting the first antibody with an antibody dilution buffer. Control slides/grids showed that little or no unspecific labelling was produced in the tissues of interest by any of the techniques employed (Fig. 7).

## Abbreviations

CNS: central nervous system; RF: Ressner's fiber; RS: Ressner's substances; SCO: subcomissural organ

## Competing interests

The authors declare that they have no competing interests.

## Authors' contributions

VSM, ORZ, TH, and JGA conceived the study and equally participated in the interpretation of the results. JMG and MC obtained the AFRU antiserum. WWN obtained the RS-K10 antiserum. JMG and WWN contributed to the interpretation of the data. BA and TH developed and tested the protocol of immunogold staining. VSM and ORZ carried out immunostaining reactions. VSM wrote the first draft of the manuscript. All authors read and approved the final manuscript.
